# PD37 Balancing The Triple Bottom Line For Health Technology Assessment: How To Support Sustainable Quality Improvement In Surgery

**DOI:** 10.1017/S0266462325101700

**Published:** 2025-12-29

**Authors:** Melissa Pegg

## Abstract

**Introduction:**

The United Nations 2030 Agenda for Sustainable Development aims to provide a “shared blueprint for peace and prosperity for people and planet, now and into the future.” Improving the quality of patient care goes end-to-end with sustainability across surgical care pathways, including pre-, intra-, and postoperative surgical optimization and maximal patient benefit. The Green Surgery report provides initiatives and recommendations to reduce the adverse impact of surgical care on the environment.

**Methods:**

Representatives from a consortium of 23 global healthcare professional organizations met every three months to provide different perspectives on sustainable surgical care practices and technologies. Consortium members were consulted throughout the development of the report. Real-world evidence was collected from case studies focusing on triple bottom line outcomes. Surgical care products and practices were evaluated using the following criteria: prevention, resource optimization, low carbon alternatives, patient optimization, leaner pathways, and patient empowerment. Data were grouped and synthesized to spotlight health technology environmental impact along the surgical care pathway. Recommendations to stakeholders based on short- and long-term objectives.

**Results:**

Surgical care is a major area of resource consumption, with the annual carbon footprint of surgical care in the UK estimated at 5.7 million tons of carbon dioxide equivalent. Carbon hotspots in the operating theatre include anesthetic gases, energy, and products (particularly single use). This report signaled the need for sustainable products and services from industry, encouraging wider systems change toward disease prevention and health promotion, alongside equitable access to high quality care. The emergence of artificial intelligence and its anticipated application within healthcare settings needs careful evaluation, as it may lead to overdiagnosis and excessive use of healthcare and associated carbon.

**Conclusions:**

This report highlighted emerging evidence that can support the transition to green surgical care. Bringing about real-world change relies upon coordinated action from all individuals and organizations influencing surgical care. It is recognized that the triple bottom line framework has some limitations, including in evaluation (data for each pillar), practical application (competing healthcare priorities), and strategic investment prioritization.

